# Risk factors of mortality in patients with rheumatoid arthritis-associated interstitial lung disease: a single-centre prospective cohort study

**DOI:** 10.1186/s13075-024-03362-1

**Published:** 2024-07-19

**Authors:** Yeo-Jin Song, Hyoungyoung Kim, Soo-Kyung Cho, Hye Won Kim, Chaewhi Lim, Eunwoo Nam, Chan-Bum Choi, Tae-Hwan Kim, Jae-Bum Jun, Sang-Cheol Bae, Dae Hyun Yoo, Su Jin Hong, Seung-Jin Yoo, Youkyung Lee, Yoon-Kyoung Sung

**Affiliations:** 1 Department of Rheumatology, Hanyang University Hospital for Rheumatic Diseases, Seoul, Republic of Korea; 2 Hanyang University Institute for Rheumatology Research, Seoul, Republic of Korea; 3 Department of Radiology, Hanyang University College of Medicine, Hanyang University Guri Hospital, Guri, Republic of Korea; 4 Department of Radiology, Hanyang University College of Medicine, Hanyang University Hospital, Seoul, Republic of Korea

**Keywords:** Rheumatoid arthritis, Interstitial lung disease, Mortality

## Abstract

**Objectives:**

To determine the risk factors for mortality in Korean patients with rheumatoid arthritis (RA)-associated interstitial lung disease (ILD) in comparison to patients with RA but without ILD (RA-nonILD).

**Methods:**

Data were extracted from a single-centre prospective cohort of RA patients with a chest computed tomography scan at an academic referral hospital in Korea. Patients with RA-ILD enroled between May 2017 and August 2022 were selected, and those without ILD were selected as comparators. The mortality rate was calculated, and the causes of each death were investigated. We used Cox proportional hazard regression with Firth’s penalised likelihood method to identify the risk factors for mortality in patients with RA-ILD.

**Results:**

A total of 615 RA patients were included: 200 with ILD and 415 without ILD. In the RA-ILD group, there were 15 deaths over 540.1 person-years (PYs), resulting in mortality rate of 2.78/100 PYs. No deaths were reported in the RA-nonILD group during the 1669.9 PYs. The primary causes of death were infection (nine cases) and lung cancer (five cases), with only one death attributed to ILD aggravation. High RA activity (adjusted HR 1.87, CI 1.16–3.10), baseline diffusing capacity for carbon monoxide (DLCO) < 60% (adjusted HR 4.88, 95% CI 1.11–45.94), and usual interstitial pneumonia (UIP) pattern (adjusted HR 5.13, 95% CI 1.00–57.36) were identified as risk factors for mortality in RA-ILD patients.

**Conclusion:**

Patients with RA-ILD have an elevated risk of mortality compared with those without ILD. Infection-related deaths are the main causes of mortality in this population. High RA activity, low DLCO, and the UIP pattern are significantly associated with the mortality in patients with RA-ILD.

**Supplementary Information:**

The online version contains supplementary material available at 10.1186/s13075-024-03362-1.

## Introduction

Rheumatoid arthritis (RA) is a chronic inflammatory autoimmune disease that frequently accompanies extra-articular manifestations such as rheumatoid nodules; vasculitis; and pulmonary, cardiovascular, and haematological diseases [1, 2]. Interstitial lung disease (ILD) was first reported in 1984 [3] and is a serious complication in patients with RA that leads to increased mortality [4, 5]. Although RA-associated ILD (RA-ILD) was previously thought to be the result of prolonged RA, it is currently understood that ILD can develop throughout the course of the disease or even before RA diagnosis [5, 6]. There is a lack of evidence on the optimal treatment for patients with RA-ILD. Physicians must consider the effect of disease-modifying antirheumatic drugs (DMARDs) on ILD in patients with RA-ILD, which narrows the treatment options and makes it difficult to control RA activity [7].

Patients with RA have an elevated risk of mortality compared with the general population [8, 9]. A large population-based cohort study conducted in Korea demonstrated a total age- and sex-adjusted standardised mortality ratio of 1.65 (95% confidence interval [CI] 1.44–1.87) in patients with RA [9]. Notably, among patients with RA, those with ILD are particularly vulnerable to mortality, with ILD identified as the leading cause of death following cardiovascular disease [8]. A substantial disparity in the risk of mortality was observed between RA-ILD and RA-nonILD patients, with RA-ILD patients having a 2 to 10 times higher risk of death [4, 5]. Specifically, the hazard rate ratio peaked at 10.4 (95% CI 5.9–18.2) during the first month following RA-ILD diagnosis. Furthermore, the stratified analysis found the risk to be higher in patients diagnosed with RA prior to the development of ILD [5].

Numerous studies have focused on identifying predictors of mortality in RA-ILD patients to better understand the disease course and enhance clinical management [4, 10–15]. Demographic factors, such as older age, male sex, and smoking history, have been recognised as prognostic indicators associated with mortality in patients with RA-ILD [4, 10–12]. Additionally, pulmonary physiological measures, including low percentage of predicted diffusing capacity for carbon monoxide (DLCO) and low percentage of predicted forced vital capacity (FVC), have emerged as predictors of ILD progression [11–15]. Computed tomography (CT) findings have also been explored, with the usual interstitial pneumonia (UIP) pattern identified as a marker for poor prognosis compared to other types, such as the non-specific interstitial pneumonia (NSIP) pattern [10–12]. However, the impact of CT patterns on mortality remains controversial, as some studies have not found any significant difference [13, 15]. Additionally, the association between seropositivity and mortality in RA-ILD patients has yielded inconsistent results [10, 12].

Despite advancements in the management of RA-ILD, there are still significant gaps in the current understanding of this condition and optimal strategies for its management [16–18]. In the present study, we aimed to investigate the causes of death and identify the risk factors associated with mortality in Korean patients with RA-ILD in comparison to patients with RA but without ILD (RA-nonILD) using data from a prospective cohort.

## Methods

### Study population

The Hanyang University Medical Centre Arthritis Network-ILD Screening and Management (HUMANISM) cohort is a multidisciplinary prospective cohort of patients with RA at an academic referral hospital in Korea, established in May 2017 (clinicaltrials.gov NCT03099525) [19]. The HUMANISM cohort included patients aged 19 years or older, who met the 1987 American College of Rheumatology (ACR) or the 2010 ACR/European Alliance of Associations for Rheumatology (EULAR) classification criteria for RA and signed an informed consent. Additionally, patients were required to undergo a chest CT scan within the two years prior to joining the study and provide written consent. The enroled patients were assigned to either the RA-ILD or the RA-nonILD group according to the results of chest CT scans evaluated by radiologists and rheumatologists. Consensus between the radiologists and rheumatologists was reached if a disagreement in the interpretation of the chest CT scans was noted. Patients diagnosed with other autoimmune diseases (such as inflammatory myositis, systemic sclerosis, systemic lupus erythematosus, and mixed connective tissue disease) were excluded. Patients with asbestosis or a history of lobectomy, pneumonectomy, or radiation therapy were also excluded. Patients enroled in the HUMANISM cohort between May 2017 and August 2022 were included.

### Data collection

Demographic and clinical information of the patients was collected at enrolment. Demographic features including age, sex, body mass index, socioeconomic status, and smoking status were obtained by interviewing the patient. Clinical characteristics included comorbidities, medication use, RA disease activity, and patient-reported outcomes, such as the Health Assessment Questionnaire-Disability Index (HAQ-DI), EuroQol-5-Dimensions questionnaire (EQ-5D), and the patient’s global assessment values. Clinical information was obtained by interviewing the participants and reviewing their medical records. Comorbidities within 3 years of enrolment were investigated, and the Charlson Comorbidity Index (CCI) score was used to assess comorbidities [20]. Connective tissue diseases were excluded from the calculation of the CCI score in this study because all patients had RA. Laboratory results, including erythrocyte sedimentation rate (ESR), C-reactive protein (CRP), rheumatoid factor (RF), and anti-citrullinated protein antibody (ACPA) levels, were also collected. Patients in the RA-ILD group underwent chest CT scan and pulmonary function tests (PFTs). The interpretation of chest CT scans performed by radiologists and the type of ILD were determined based on the official ATS/ERS/JRS/ALAT clinical practice guidelines [21].

Annual follow-ups were conducted to investigate each participant’s clinical information in both groups, and observations were terminated if the patient died, was lost to follow-up, or reached June 2023. As this was a completely observational study, no intervention other than treatment determined by the physician in a real-world clinical setting was applied.

### Statistical analyses

We compared the baseline characteristics between the RA-ILD and RA-nonILD groups using the chi-square test or Fisher’s exact test for categorical variables and the Mann–Whitney U test for continuous variables. The clinical characteristics of patients with RA-ILD, including the results of PFTs and CT findings, were described. The mortality rate was calculated as cases per 100 person-years (PYs) and the causes of death were investigated. RA-ILD and RA-nonILD mortality were visualised using the Kaplan-Meier survival curve. Cox proportional hazard regression with Firth’s penalised likelihood method, which is robust for sparse data, was performed to compare mortality between the two groups after adjusting for covariates. To identify the risk factors for mortality in patients with RA-ILD, we used Cox proportional hazard regression with Firth’s penalised likelihood. The multivariable regression model included variables that were significant in the univariable regression analysis.

All analyses were performed using SAS^®^ 9.4 software (SAS Institute, Cary, NC, USA) or R software version 3.4.2 (R Foundation for Statistical Computing, Vienna, Austria). Missing values were excluded from the analyses without imputation, and the results were considered statistically significant when the *P*-value was < 0.05.

## Results

### Baseline characteristics of the study population

**Fig. 1:**
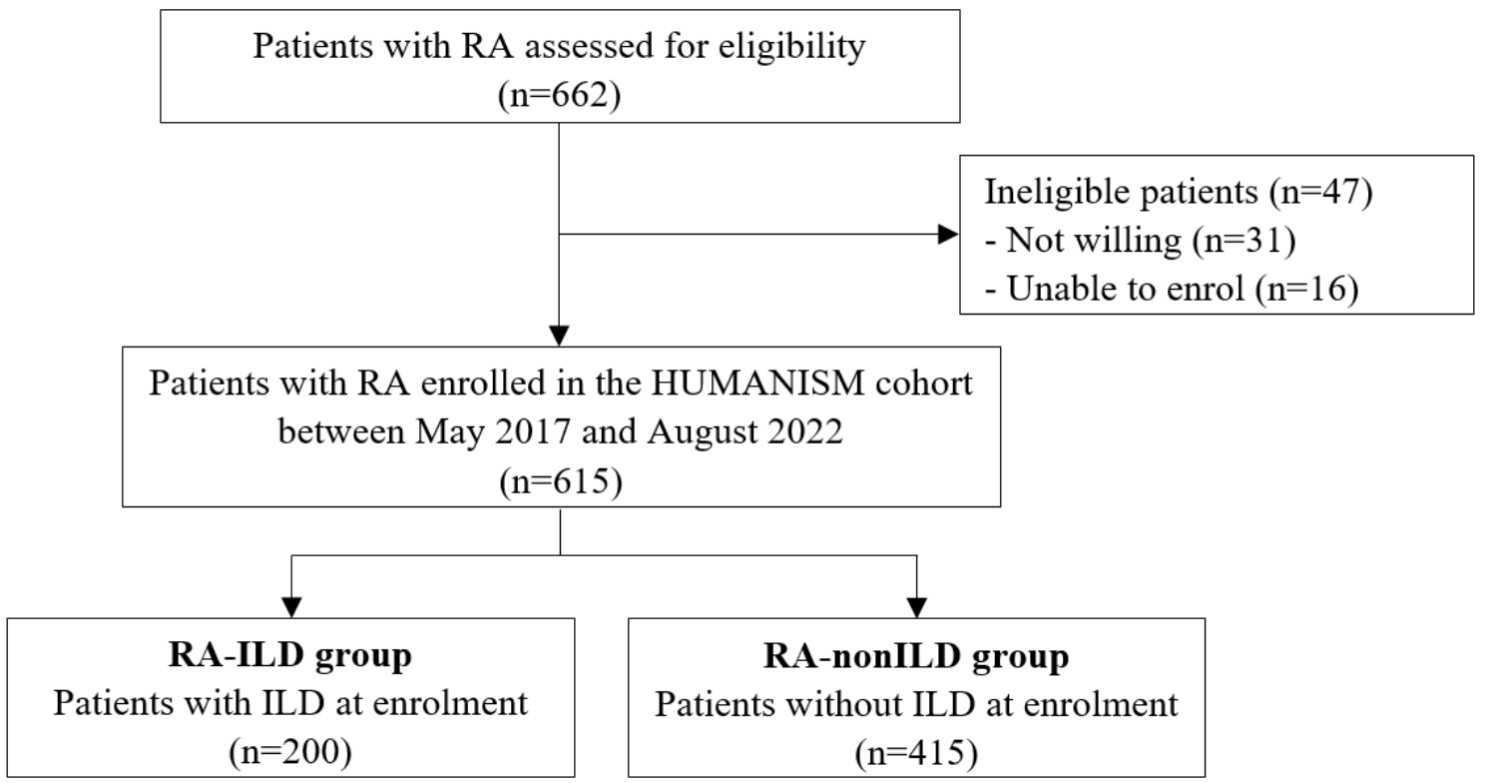
Flow-chart of patient selection. RA, rheumatoid arthritis; HUMANISM, Hanyang University Medical Centre Arthritis Network Interstitial Lung Disease Screening and Management; ILD, interstitial lung disease

Among the 662 eligible patients with RA, patient who were not willing to or unable to participate the study were excluded (Fig. 1). The numbers of patients lost to follow-up in each group are presented in Supplementary Fig. 1 [see Additional File 1].

A total of 615 patients were included in the analysis: 200 in the RA-ILD group and 415 in the RA-nonILD group. The median age of the study population was 61.2 years, and 21.3% were male patients (Table 1).

**Table 1:** Baseline characteristics of the study population at enrolment

Variables	Total (*n* = 615)		RA-ILD (*n* = 200)		RA-nonILD (*n* = 415)		*P*
Sex, male	131	(21.3)	68	(34.0)	63	(15.2)	< 0.001
Age, years	61.2	(53.8–68.2)	66.8	(60.8–73.0)	58.6	(51.8–64.6)	< 0.001
Duration of RA, years (*n* = 521, 120, 401)	8.8	(4.4–13.6)	6.9	(3.2–13.2)	9.4	(5.1–13.7)	0.001
Body mass index, kg/m^2^	23.1	(20.8–25.3)	23.5	(21.0–25.8)	23.1	(20.7–25.1)	0.082
Smoking history	148	(24.1)	71	(35.5)	77	(18.6)	< 0.001
Family history of pulmonary diseases	81	(13.2)	21	(10.5)	60	(14.5)	0.342
History of pulmonary tuberculosis	90	(14.6)	18	(9.0)	72	(17.4)	0.006
CCI score^a^	0	(0–0)	0	(0–1)	0	(0–0)	< 0.001
Seropositivity	594	(96.6)	193	(96.5)	401	(96.6)	0.936
RA activity							
ESR	25	(12–41)	30	(15–51)	21	(11–37)	< 0.001
DAS28-ESR	3.3	(2.6–4.3)	3.4	(2.7–4.4)	3.2	(2.5–4.2)	0.020
Physician’s global assessment	20	(20–40)	30	(20–40)	20	(20–40)	0.168
Patient-reported outcomes							
HAQ-DI	0.6	(0.2–1.2)	0.9	(0.4–1.5)	0.5	(0.1–1.1)	< 0.001
EQ-5D	0.8	(0.7–0.8)	0.8	(0.7–0.8)	0.8	(0.8–0.9)	< 0.001
Patient’s global assessment^b^	33.9	± 21.9	37.0	± 23.3	32.4	± 21.0	0.029
Extra-articular manifestations of RA^c^ (*n* = 522, 120, 402)	30	(4.9)	5	(2.5)	25	(6.0)	0.057
Pulmonary function test							
FVC, % of predicted (*n* = 190)			80.6	± 16.0			
DLCO, % of predicted (*n* = 178)			56.2	± 14.2			
Predominant type of ILD (*n* = 197)							
UIP			131	(66.5)			
Definite UIP			83	(42.1)			
Probable UIP			48	(24.4)			
NSIP			42	(21.3)			
OP			2	(1.0)			
Others^d^			22	(11.2)			

Categorical data were presented as numbers with percentages (%) and compared using the chi-square or Fisher’s exact test, and continuous variables were presented as median (IQR) or mean ± SD and compared using the Mann–Whitney U test

^a^ All patients had rheumatoid arthritis; thus, connective tissue diseases were excluded from the CCI calculation in this study

^b^ The patient’s global assessment is represented as mean ± SD instead of median (Q_1_–Q_3_) = 30 (20–50) and median (range) = 30 (0–90) in both groups

^c^ Extra-articular manifestations of RA include pericarditis, pleuritis, Felty’s syndrome, vasculitis, scleritis, or nephritis

^d^ A total of 21 patients who were indeterminate and one patient with RB-ILD were included

RA, rheumatoid arthritis; ILD, interstitial lung diseases; COPD, chronic obstructive pulmonary disease; CCI, Charlson Comorbidity Index; ESR, erythrocyte sedimentation rate; DAS, disease activity score; HAQ-DI, Health Assessment Questionnaire-Disability Index; EQ-5D, EuroQol-5 Dimensions questionnaire; FVC, forced vital capacity; DLCO, diffusing capacity for carbon monoxide; UIP, usual interstitial pneumonia; NSIP, non-specific interstitial pneumonia; OP, organizing pneumonia; IQR, interquartile range (Q_1_–Q_3_); RB-ILD, Respiratory bronchiolitis interstitial lung disease

Patients in the RA-ILD group were older (66.8 years vs. 58.6 years, *P* < 0.001), and the proportion of male patients was higher in the RA-ILD group (34.0% vs. 15.2%, *P* < 0.001). There were significantly more ever-smokers in the RA-ILD group (35.5% vs. 18.6%, *P* < 0.001), and patients with RA-ILD had more comorbidities. Disease activity of RA estimated by the Disease Activity Score 28 (DAS28)-ESR was higher in the RA-ILD than the RA-nonILD group (3.4 vs. 3.2, *P* = 0.020).

For patients with RA-ILD who underwent PFTs at enrolment, the mean predicted percentage of FVC was 80.6%, and the mean predicted percentage of DLCO was 56.2% (Table 1). The median time between chest CT scans and enrolment was 91 days. Among the CT scans, the most prevalent ILD pattern observed was UIP (66.5%), consisting of definite UIP (42.1%) and probable UIP (24.4%). Patients with NSIP pattern accounted for 21.3%. Reticulation (97.5%) was the most common CT finding, followed by traction bronchiectasis (89.3%).

The medication use patterns of the two groups are presented in Table 2. Methotrexate and leflunomide were prescribed less frequently in the RA-ILD group. Tumour necrosis factor (TNF) inhibitors were used more commonly in the RA-nonILD group, whereas non-TNF inhibitors were used more in the RA-ILD group. Patients with RA-ILD were more frequently treated with higher doses of oral glucocorticoids.

**Table 2:** Medication use of the study population at enrolment

Variables	Total (*n* = 615)		RA-ILD (*n* = 200)		RA-nonILD (*n* = 415)		*P*
Conventional synthetic DMARDs							
Methotrexate	455	(74.0)	113	(56.5)	342	(82.4)	< 0.001
Hydroxychloroquine	159	(25.9)	81	(40.5)	78	(18.8)	< 0.001
Sulfasalazine	153	(24.9)	75	(37.5)	78	(18.8)	< 0.001
Leflunomide	113	(18.4)	11	(5.5)	102	(24.6)	< 0.001
Tacrolimus	75	(12.2)	36	(18.0)	39	(9.4)	0.002
Immunosuppressants							
Azathioprine	10	(1.6)	4	(2.0)	6	(1.5)	0.735
Cyclosporine	7	(1.1)	5	(2.5)	2	(0.5)	0.040
Targeted therapy							
TNF inhibitors	76	(12.4)	13	(6.5)	63	(15.2)	0.002
Adalimumab	34	(5.5)	10	(5.0)	24	(5.8)	0.691
Etanercept	31	(5.0)	2	(1.0)	29	(7.0)	0.002
Golimumab	10	(1.6)	1	(0.5)	9	(2.2)	0.179
Infliximab	1	(0.2)	0	(0.0)	1	(0.2)	0.999
Non-TNF inhibitors	41	(6.7)	25	(12.5)	16	(3.9)	< 0.0001
Abatacept	24	(3.9)	18	(9.0)	6	(1.5)	< 0.001
Tocilizumab	12	(2.0)	4	(2.0)	8	(1.9)	0.999
Rituximab	5	(0.8)	3	(1.5)	2	(0.5)	0.336
JAK inhibitors	19	(3.1)	2	(1.0)	17	(4.1)	0.038
Tofacitinib	18	(2.9)	1	(0.5)	17	(4.1)	0.013
Baricitinib	1	(0.2)	1	(0.5)	0	(0.0)	0.325
Other medications							
NSAIDs	337	(54.8)	117	(58.5)	220	(53.0)	0.200
Oral glucocorticoid	209	(34.0)	113	(56.5)	96	(23.1)	< 0.001
Oral glucocorticoid dose, mg/day^a^	2.5	(2.5–5)	5.0	(2.5–5)	2.5	(2.5–5)	< 0.001

Categorical data were presented as numbers with percentages (%) and compared using the chi-square or Fisher’s exact test, and continuous variables were presented as median (IQR) and compared using the Mann–Whitney U test

^a^ Regularly prescribed oral glucocorticoids were considered in the calculation, and the average was presented as the prednisolone-equivalent dose

RA, rheumatoid arthritis; ILD, interstitial lung diseases; DMARD, disease-modifying antirheumatic drugs; TNF, tumour necrosis factor; JAK, Janus kinase; NSAID, non-steroidal anti-inflammatory drug; IQR, interquartile range (Q1–Q3)

### Mortality and causes of death

**Fig. 2:**
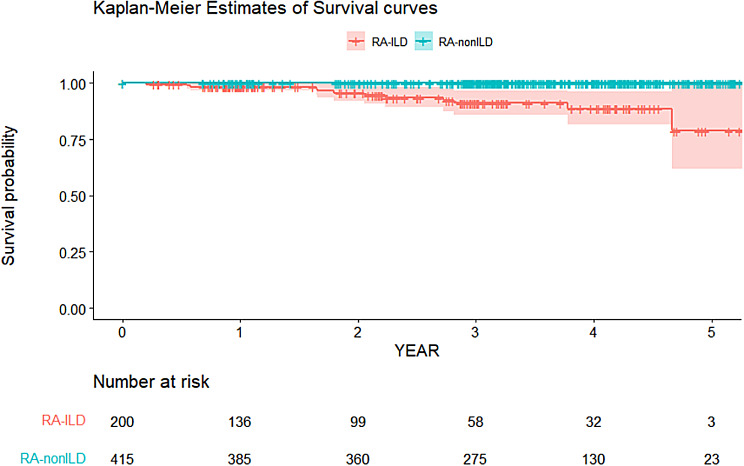
Kaplan-Meier survival curves in RA patients with ILD versus nonILD. The shaded part represents the 95% confidence interval for the survival probability. Each vertical drop in the curve indicates one or more events (death). Right-censored cases are indicated by vertical marks on the curves at the censoring time. RA, rheumatoid arthritis; ILD, interstitial lung disease

Up until June 2023, 15 deaths occurred in the RA-ILD group during the 540.1 person-years (PYs) (median 2.6, interquartile range [IQR] 1.2–4.1 years), and the mortality rate was 2.78/100 PYs (95% CI 1.56–4.54). No deaths were observed in the RA-nonILD group during the 1669.9 PYs (median 4.2, IQR 3.6–4.9 years). The Kaplan-Meier survival curve between the RA-ILD and RA-nonILD groups is presented in Fig. 2. After adjusting for age, sex, smoking history, and baseline DAS28-ESR, the hazard ratio (HR) in the RA-ILD group was found to be significantly higher than that in the RA-nonILD group based on a Cox proportional hazard regression with Firth’s penalised likelihood method (Supplementary Table 1: adjusted HR 43.68, 95% CI 4.92–5791.23) [see Additional File 1].

The detailed characteristics of the deceased patients are presented in Supplementary Table 2 [see Additional File 1]. The most common cause of death was infection (nine cases), followed by lung cancer (five cases). Only one patient died due to ILD aggravation. All of the deceased patients had a predominant UIP pattern except one with an NSIP pattern.

### Risk factors for mortality in RA patients with ILD

In the multivariable regression analysis for patients with RA-ILD, high RA activity assessed by DAS28-ESR (adjusted HR 1.87, 95% CI 1.16–3.10) and baseline DLCO < 60% of the predicted value (adjusted HR 4.88, 95% CI 1.11–45.94) were significant risk factors for mortality after adjusting for other confounding factors (Table 3). In addition, the UIP pattern (adjusted HR 5.13, 95% CI 1.00–57.36) was a marginally significant risk factor for mortality.

**Table 3:** Risk factors for mortality in patients with RA-ILD

Variables	Univariable analysis (*n* = 200)		Multivariable analysis^*^ (*n* = 178)	
	Unadjusted HR (95% CI)	***P***	Adjusted HR (95% CI)	***P***
Age at enrolment, years	1.04 (0.99–1.10)	0.124	1.05 (0.99–1.13)	0.132
Sex, male (ref. female)	1.95 (0.69–5.48)	0.194	2.76 (0.83–9.87)	0.098
Smoking (ref. never smoking)	1.25 (0.44–3.52)	0.662		
Duration of RA, years	1.04 (0.98–1.10)	0.235		
Body mass index	0.92 (0.78–1.08)	0.298		
CCI score	0.82 (0.36–1.87)	0.631		
Seropositivity	0.35 (0.06–2.03)	0.285		
DAS28-ESR	1.43 (1.02–1.99)	0.045	1.87 (1.16–3.10)	0.011
DLCO < 60% of predicted (*n* = 178)	6.7 (1.15–38.94)	0.006	4.88 (1.11–45.94)	0.035
FVC < 80% of predicted (*n* = 190)	2.68 (0.92–7.82)	0.053		
UIP pattern (*n* = 197)	6.14 (1.08–34.97)	0.008	5.13 (1.00–57.36)	0.049

Unadjusted and adjusted HRs were computed using Cox proportional hazard regressions with Firth’s penalised likelihood method

^a^ The multivariable regression model included age, sex, and variables significant in the univariable regression analysis

RA, rheumatoid arthritis; IRR, incidence rate ratio; CI, confidence interval; ILD, interstitial lung disease; CCI, Charlson Comorbidity Index; DAS, disease activity score; ESR, erythrocyte sedimentation rate; DLCO, diffusing capacity for carbon monoxide; FVC, forced vital capacity; UIP, usual interstitial pneumonia

## Discussion

In this single-centre prospective cohort study, we identified the differing characteristics between RA-ILD and RA-nonILD patients. Deaths occurred only in the RA-ILD group over 2.6 years of observation, whereas none occurred in the RA-nonILD group over the 4.2-year observation period. The most common cause of death was infection, and the risk factors for mortality among RA-ILD patients were high RA activity, baseline DLCO < 60% of the predicted value, and the UIP pattern on chest CT scan.

The characteristics of patients with RA-ILD were markedly different from those of patients with RA without ILD. The proportion of male patients was higher in the RA-ILD group than in the RA-nonILD group. Patients with RA-ILD also had more comorbidities; however, a history of pulmonary tuberculosis was more common in the RA-nonILD group. In our study, the proportion of patients with a history of pulmonary tuberculosis was high. According to a previous study on the comorbidities in Korean patients with RA, 8.6% of patients with RA had pulmonary tuberculosis [22]. In addition, our previous study revealed that the latent tuberculosis infection positivity rate was 26.5% in patients with RA initiating targeted therapy at a tertiary referral hospital in Korea [23]. The information about the history of pulmonary tuberculosis was obtained by interviewing patients in addition to reviewing medical records; therefore, the prevalence of pulmonary tuberculosis could be overestimated. Nevertheless, none of the patients had active pulmonary tuberculosis at enrolment.

One intriguing finding of this study was that deaths occurred exclusively in the RA-ILD group, which made a direct comparison of the mortality rates between the two groups not possible. However, this observation suggests a potential association between ILD and an increased risk of mortality in patients with RA. It is important to acknowledge the differences in the baseline characteristics between the two groups, such as age, smoking status, comorbidities, and RA activity, as these factors may have influenced the outcomes. Although a comprehensive analysis adjusting for these variables would provide a more accurate understanding of the impact of ILD on mortality, we were unable to conduct further analysis due to the absence of deaths in the RA-nonILD group.

The primary cause of death in this study was infection, with pneumonia being the most prevalent cases of urosepsis and biliary sepsis. Patients with RA have an increased susceptibility to infections compared to the general population. This susceptibility can be attributed to immunological dysfunction, comorbidities, and the use of immunomodulatory drugs [24]. Considering that no deaths occurred in the RA-nonILD group, RA-ILD may be a potential risk factor for developing fatal pneumonia. It is worth noting that, of the 15 deceased patients, six had received targeted therapy, and five of them had their deaths attributed to infection. This observation raises the possibility of an increased risk of infection associated with the targeted therapy. However, it is important to note the high RA disease activity levels that necessitated the use of targeted therapy in these patients.

The second most common cause of death in patients with RA-ILD was cancer, with all five cases being lung cancer. Patients with RA have an approximately 1.5-fold higher risk of developing lung cancer than the general population [25, 26]. The hypothesis is that lung cancer and RA share a common risk factor: smoking [27]. In addition, ILD is considered as a possible risk factor for lung cancer, with a recent retrospective cohort study conducted in China reporting that approximately 3% of patients with ILD were diagnosed with lung cancer [27, 28]. In our study, all five patients who died of lung cancer were smokers.

Higher RA activity and the UIP pattern were significant risk factors for mortality in patients with RA-ILD. The UIP pattern has been recognised as a poor prognostic factor compared with other patterns, such as the NSIP pattern [11, 12, 15], and our study showed similar trends. However, it is important to investigate whether the UIP pattern association with poor outcomes is truly due to an ILD flare-up or other factors, such as infection or malignancy. Further detailed analyses are required to confirm this hypothesis.

One of the risk factors for mortality in the RA-ILD group was a baseline DLCO < 60% of the predicted value. The importance of pulmonary physiology in the prognosis of patients with RA-ILD has been previously reported. According to previous studies, low baseline DLCO was related to the risk of ILD progression and mortality [12, 13, 29]. Low baseline FVC is also known to be a risk factor for mortality in patients with RA-ILD [11, 12, 15, 29]; however, this was not true in our study. PFT results correlate more significantly with the extent of lung involvement than with the ILD pattern [30]. This indicates that the importance of PFT results and CT findings may differ when evaluating the prognosis of patients with RA-ILD as individuals or in a group.

Considerations when prescribing DMARDs for patients with RA-ILD are more complex compared to those without ILD. Methotrexate is the first-line treatment recommended for patients with RA; however, its effects on ILD remain controversial [31, 32]. An inception cohort study of patients with early RA showed that methotrexate treatment reduced the risk and delayed the incidence of ILD [33]. In contrast, leflunomide is not recommended for patients with RA-ILD because of its association with an increased risk of the development and/or exacerbation of ILD [34]. However, a meta-analysis of RCTs found no evidence of increased respiratory adverse events in RA patients [35]. In our study, the proportion of patients with RA-ILD treated with methotrexate or leflunomide was significantly lower than that of RA-nonILD patients. Regarding targeted therapy, abatacept was most frequently used in the RA-ILD group and was associated with a slower deterioration of ILD and a lower risk of infection than TNF inhibitors [36, 37].

The 2023 ACR guidelines for the treatment of ILD in individuals with systemic autoimmune rheumatic disease were recently announced, and a summary was posted [38]. Mycophenolate, azathioprine, and rituximab are recommended as first-line therapies for patients with RA-ILD according to the guidelines. However, mycophenolate mofetil is not approved for patients with RA in Korea. In addition, the prescription rates for azathioprine and rituximab were low in our study. In Korea, rituximab is approved only for patients who have an inadequate response to other targeted therapies (TNF inhibitors, non-TNF inhibitors, or Janus kinase inhibitors). Our results are expected to serve as important evidence demonstrating the discrepancies between the new ACR guidelines and medication use in Korean patients with RA-ILD.

This study had several strengths. First, we used data from a well-established multidisciplinary prospective cohort. In particular, we improved the reliability of CT readings by reaching a consensus through discussions between rheumatologists and radiologists. Second, we identified the risk factors for mortality in patients with RA-ILD, which still have an unmet need for clinical studies in Korea.

This study had several limitations. First, this was a single-centre study, which could be questioned for generalisability. However, our hospital contains the largest rheumatology centre in the nation, drawing patients from across the country. In addition, more detailed information could be collected by interviewing patients directly or through medical records because the study was performed at a single institution. Second, all patients in the RA-nonILD group survived the observation period; therefore, it was not possible to compare the mortality risk between the two groups. Further studies with longer observation periods are necessary. Third, some ILD patients and the majority of nonILD patients did not undergo PFTs; therefore, we could not compare the PFT results between the two groups. However, there were only 12 patients without PFTs in the ILD group, and the number of RA-ILD patients with PFTs was considered sufficient for multivariable analysis.

In conclusion, patients with RA-ILD had an increased risk of mortality compared to RA-nonILD patients. The main causes of death were infections, especially pneumonia, and lung cancer. Risk factors for mortality in patients with RA-ILD are high RA disease activity, low baseline DLCO, and the UIP pattern. Further studies with longer observation periods are needed to clarify the increased incidence of mortality in RA-ILD patients compared to that in RA-nonILD patients.

### Electronic supplementary material

Below is the link to the electronic supplementary material.

Supplementary Material 1

## Data Availability

No datasets were generated or analysed during the current study.
