# Supplementary material for: Risk factors of mortality in patients with rheumatoid arthritis-associated interstitial lung disease: a single-centre prospective cohort study
Source: Arthritis Res Ther. 2024 Jul 19;26:137. doi: 10.1186/s13075-024-03362-1 (PMC11264792; doi:10.1186/s13075-024-03362-1)
Supplement: Supplementary file 1 — Supplementary Material 1 [file 13075_2024_3362_MOESM1_ESM.pdf]

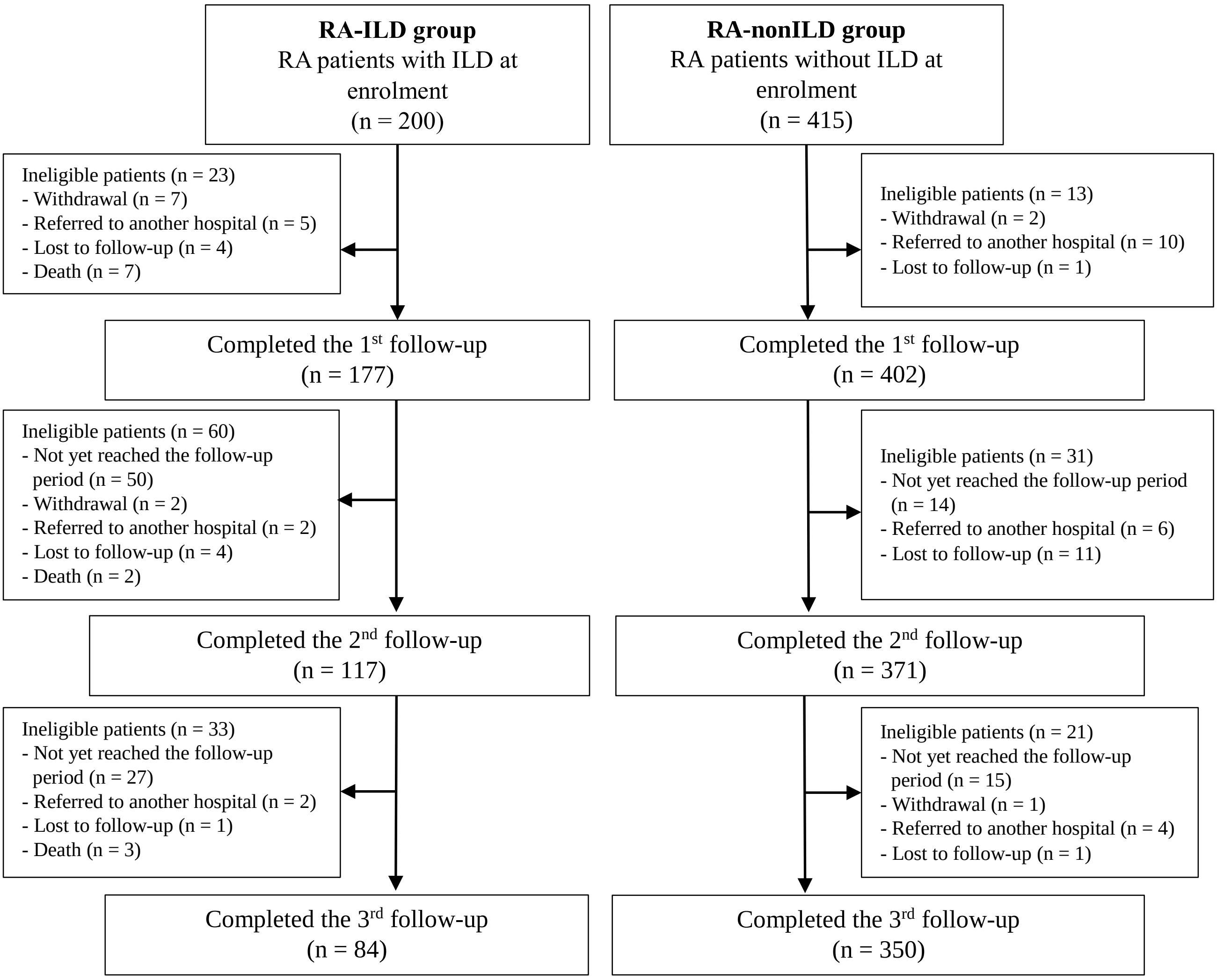

Supplementary Figure 1. Follow-up of the study population until June 2023

RA, rheumatoid arthritis; ILD, interstitial lung disease.

Supplementary Table 1. Comparison of survivals in RA patients with ILD versus nonILD

Variables	Univariable analysis		Multivariable analysis*	
	Unadjusted HR (95% CI)	*P*	Adjusted HR (95% CI)	*P*
Age at enrolment, years	1.11 (1.04–1.19)	0.001	1.03 (0.95–1.11)	0.503
Sex, male (ref. female)	7.71 (2.54–26.81)	<0.001	22.38 (2.84–169.48)	0.005
Smoking history (ref. never)	3.63 (1.19–11.08)	0.025	0.16 (0.03–1.03)	0.053
Duration of RA, years	1.00 (0.91–1.07)	0.934		
Body mass index	0.93 (0.78–1.11)	0.439		
CCI score	1.35 (0.54–2.52)	0.464		
Seropositivity	0.29 (0.07–2.64)	0.218		
DAS28-ESR	1.67 (1.10–2.49)	0.018	1.74 (1.14–2.64)	0.011
**ILD (ref. non-ILD)**	**88.64 (11.61–11372.79)**	**<0.001**	**43.68 (4.92–5791.23)**	**<0.001**

Adjusted HRs were computed using Cox proportional hazard regression with Firth's penalised likelihood method after adjusting for age, sex, smoking history, and baseline DAS28-ESR.

RA, rheumatoid arthritis; ILD, interstitial lung disease; HR, hazard ratio; CI, confidence interval; CCI, Charlson comorbidity index; DAS, disease activity score; ESR, erythrocyte sedimentation rate.

Supplementary Table 2. Characteristics of deceased patients with RA-ILD

	Sex	Age at death	Cause of death	Duration of RA, years	Duration of ILD, years	Smoking	DMARDs[a]	Targeted therapy[a]	Type of ILD	FVC % of predicted at baseline	DLCO % of predicted at baseline
**Case 1**	M	56	Cancer (NSCLC)	6.6	5.8	current smoker	MTX, SSZ, TAC	-	definite UIP	92.8	46.5
**Case 2**	M	64	Cancer (NSCLC)	2.3	3.1	current smoker	MTX, HCQ, SSZ, TAC	-	definite UIP	90.5	48.0
**Case 3**	M	66	Infection (Pneumonia)	31.8	4.6	ex-smoker	MTX	Adalimumab Tocilizumab Abatacept	definite UIP	70.9	50.5
**Case 4**	M	67	Cancer (SCLC)	7.2	7.2	current smoker	LEF, SSZ, BUC		definite UIP	43.1	47.7
**Case 5**	F	72	Infection (Pneumonia)	5.9	5.9	non-smoker	MTX, SSZ, BUC	Tocilizumab Abatacept	NSIP	65.1	38.1
**Case 6**	F	74	Infection (Pneumocystic pneumonia)	2.1	2.1	non-smoker	MTX, HCQ, SSZ	Abatacept	probable UIP	58.4	59.1
**Case 7**	M	75	Acute exacerbation of ILD	0.4	1.0	non-smoker	HCQ, SSZ, BUC		definite UIP	51.0	29.1
**Case 8**	F	76	Infection (COVID-19 pneumonia)	30.5	17.3	non-smoker	HCQ, MZR, CsA	Etanercept	definite UIP	68.8	34.6
**Case 9**	M	84	Infection (Biliary sepsis)	19.7	4.8	ex-smoker	MTX, SSZ		definite UIP	61.1	31.6

Data collected at the time of death or at the last follow-up closest to death were used.

RA, rheumatoid arthritis; ILD, interstitial lung disease; DMARD, disease-modifying antirheumatic drug; FVC, forced vital capacity; DLCO, diffusing capacity for carbon monoxide; NSCLC, non-small cell lung cancer; SCLC, small cell lung cancer; COVID, coronavirus disease; MTX, methotrexate; SSZ, sulfasalazine; TAC, tacrolimus; HCQ, hydroxychloroquine; LEF, leflunomide; BUC, bucillamine; MZR, mizoribine; CsA, cyclosporine A; UIP, usual interstitial pneumonia; NSIP, non-specific interstitial pneumonia.

[a] List of medications used after ILD diagnosis.
